# Introduced non-native mangroves express better growth performance than co-occurring native mangroves

**DOI:** 10.1038/s41598-020-60454-z

**Published:** 2020-03-02

**Authors:** Fatih Fazlioglu, Luzhen Chen

**Affiliations:** 1 Key Laboratory of the Ministry of Education for Coastal and Wetland Ecosystems, College of the Environment and Ecology, Xiamen University, Xiamen, Fujian 361102 China; 2 Faculty of Arts and Sciences, Department of Molecular Biology and Genetics, Ordu University, Ordu, 52200 Turkey

**Keywords:** Forest ecology, Invasive species

## Abstract

Mangroves are salt-tolerant woody species occurring in tropical/subtropical coastal habitats. Plantation of fast-growing non-native mangrove species has been used as a tool for mangrove restoration/reforestation in several countries. However, the fast-growth ability can make recently introduced species invasive as they can possibly replace co-occurring native mangroves through expressing higher growth performance and phenotypic plasticity. Therefore, quantifying growth differences between native versus non-native mangrove species is important for forest ecology and management. In this meta-analysis, we compared the growth performance of non-native and native mangrove species pairs by analysing all available results in the literature (33 studies). We found that non-native mangrove species performed better than co-occurring native mangrove species in their introduced regions (Log response ratio = 0.51 ± 0.05) and they also expressed higher trait plasticity. Therefore, these species can be potentially invasive owing to their greater competitive advantage. However, the growth difference was diminished at higher latitudes where native mangrove species seem to perform as well as non-native mangrove species do. This is the first meta-analysis on the growth response of mangroves and it has consequential management implications. We suggest that planting of non-native mangrove species should be avoided and their spread should be monitored.

## Introduction

Mangroves are stress-tolerant species occupying coastal intertidal zones (along shores, rivers and estuaries) in the tropics and subtropics worldwide^1^. Despite their invaluable ecosystem goods and services, mangroves and the species depending on them are at an elevated extinction risk owing to coastal development, pollution, aquaculture, logging and biological invasion^2,3^. Globally, 20% of the total area of mangroves has been lost between 1980 and 2005^4^. Similarly, global climate change (e.g., elevated temperature and sea-level rise) also threaten mangrove ecosystems by creating novel environments for mangroves where new abiotic factors (e.g., higher salinity) or biotic interactions may occur (e.g., competition with recently arrived species). Therefore, the ecological consequences of global climate change on mangrove ecosystems are not straightforward.

Biological invasion is also a major threat to mangrove ecosystems. Climate change can be a driving factor regarding invasive species performance. Abrupt range shifts in invasive species are expected under global climate change across biomes^5,6^. Different components of climate change (e.g., elevated atmospheric CO_2_, increased temperatures and changes in precipitation) trigger different responses in native and non-native species and therefore build more uncertainty regarding plant invasion status and appropriate management practices^7^. For example, increased CO_2_ can favour non-native species more than native species and facilitate biological invasions^8,9^. Therefore, the effect of biological invasion and global climate change interaction remains elusive but has critical importance, especially in mangrove forests.

Several terms have been used to describe invasive species in the literature (e.g., alien, nonnative, naturalized, nonindigenous, introduced and exotic). According to the definition by Richardson *et al*. (2000) an alien plant is an introduced species due to human activities in a certain area, whereas a naturalized plant is an alien plant that can constantly reproduce. Invasive plants are naturalized plants producing an excessive amount of offspring and potentially can spread across different areas^10^. Invasive species in mangrove forests are a serious problem and the main reason for introduced species is accidental introductions^11^. However, some mangrove species were intentionally introduced to restore mangrove habitats, and later these species became invasive such as *Bruguiera* and *Rhizophora* species in the USA^12^ and *Sonneratia* species in China^13^.

Many non-native woody species have also been introduced into mangrove forests in North America, Australia and Africa^12–16^ (see Table 1 for details). According to a global database of invasive species, there are at least 751 tree and shrub species which have been considered as invasive in the world^17^. Invasive species in mangrove forests were found to prevent the establishment and growth of mangroves, alter existing native vegetation composition, prevent regeneration, and affect ecosystem functioning^11^. For example, Hawaii had no native mangroves before the introduction of *Rhizophora mangle* in 1902^15^, and currently, there are at least six mangrove species in the archipelago. These introduced mangroves might have displaced some plant species in wetlands (e.g., *Batis maritime*, *Paspalum vaginatum*, *Hibiscus tiliaceus* and *Thespesia populnea*)^18,19^.

**Table 1:** Detailed information on introduced woody species invading different mangrove ecosystems in the world.

Species list	Origin	Introduced region	Introduction year	References
**Mangrove species**				
*Avicennia marina*	Indo-Pacific	California, USA	1970	^14^
*Bruguiera gymnorhiza*	Indo-Pacific	Hawaii, USA; Florida, USA	1922 1940	^12,15^
*Excoecaria indica*	Indo-Pacific	Bangladesh	—	^92^
*Laguncularia racemosa*	Atlantic East Pacific	China	1999	^70^
*Lumnitzera racemosa*	Indo-Pacific	Florida, USA; Tonga	1960	^12^
*Nypa fruticans*	Indo-Pacific	West Africa; Caribbean	1906 —	^93,94^
*Rhizophora mangle*	Florida, USA	Hawaii, USA	1902	^15^
*Rhizophora mucronata*	Indo-Pacific	Hawaii, USA	1922	^15^
*Rhizophora racemosa*	Indo-Pacific	Saudi Arabia	—	^95^
*Rhizophora stylosa*	Indo-Pacific	French Polynesia	1937	^96^
*Sonneratia apetala*	Bangladesh	China	1985	^13^
*Sonneratia caseolaris*	Hainan, China	Shenzhen, China	—	^53^
**Mangrove associates**				
*Conocarpus erectus*	Florida, USA	Hawaii, USA	1910	^97^
*Pluchea carolinensis*	Atlantic	Pacific islands	—	^17^
*Pluchea indica*	Asia	Pacific islands	—	^17^
*Thespesia populnea*	Asia	North America, Caribbean	1928 —	^97^
**Woody species**				
*Annona glabra*	Neotropics	Australia	1912	^98^
*Casuarina equisetifolia*	Australia	North America; China; South Africa	1924 1950s —	^99–101^
*Melaleuca quinquenervia*	Australia	Florida, USA; Bahamas	1906	^102,103^
*Schinus terebinthifolius*	Neotropics	Florida, USA; Bahamas; Australia	1891 — —	^104^
*Syzygium fruticosum*	Indo-Pacific	Bangladesh	—	^92^
*Tamarix indica*	Indo-Pacific	Bangladesh	—	^92^

Note: References were listed correspondingly depending on the introduced region.

How do introduced species express better performance in growth and competition than native species and become invasive? According to the niche theory, non-native and native species those that have a similar niche cannot coexist but that have similar niche cannot coexist^20^. However, trait-based coexistence theory contradicts the niche theory^21^. According to the coexistence theory, species abundance can determine its population growth rate and competitive interactions are the main factors shaping communities^22^. Species with high abundance and distribution (e.g., common native species) may have high intraspecific trait variation because of high habitat heterogeneity. However, high trait variation can be negatively correlated with species abundance in tropical tree seedlings^23^. In other words, occupying optimal trait values (low trait variation) can contribute to a higher spread of a particular species.

High establishment of non-native mangrove species in their introduced range can be linked with reduced propagule predation (e.g., *Rhizophora mangle* in Hawaii)^24^. Moreover, natural enemies may not co-migrate with introduced non-native species when these plants expand their ranges^25^. Therefore, an invasive species may invest more in its growth and competitive ability rather than defence due to lack of enemies in the introduced regions^26^ (i.e., the evolution of increased competitive ability - EICA hypothesis). Therefore, invasive woody species can express better performance than their congeneric or con-familial relatives^27^.

Non-native woody species can also be more competitive than native woody species by expressing greater phenotypic plasticity in biomass production which can be important in tree invasions^28–30^. Similarly, high phenotypic plasticity in biomass allocation in mangroves can be very beneficial for seedling establishment^31^. High plasticity in plant traits can allow mangroves to express higher tolerances to low temperature, aridity, salinity and nutrient scarcity^32^. Therefore, mangrove species with high phenotypic plasticity are more likely to adapt climate change by expressing optimal responses in changing environmental conditions.

Functional traits are biochemical, morphological, physiological, phenological traits which can affect the performance and fitness of an individual^33,34^. Variation in a functional trait of an individual can determine the performance and fitness of the individual and its population which, in turn, can affect the community and ecosystem-level processes^35^. Therefore, scaling up from trait level responses to the ecosystem level is possible^36^. For example, specific leaf area (SLA) in woody species is positively correlated with the niche breadth of a species^37^.

Functional traits can also explain the coexistence of plants more than environmental conditions (e.g., rainfall) and evolutionary relationships (e.g., phylogenetic distance)^38^. Therefore, intraspecific trait plasticity can be a key factor to understand species fundamental niche and distribution^35^ as well as the invasive potential of a species^39^. For example, several studies indicated that higher values in some traits can be used as indicators of invasive tree species (Table 2). As a result, understanding variation in functional traits across non-native versus native mangrove species especially under changing environmental conditions caused by global climate change can be crucial for mangrove conservation.

**Table 2:** Detailed information on studies comparing invasive and native woody species in terms of growth performance.

Invasive species	Traits measured	Key traits for invasiveness	Region	Reference
18 tree and 9 shrub species	RGR, NAR, LAR, LMR, SLA	RGR and SLA	Southwest USA	^56^
57 tree or shrub species	SLA, height, flowering duration, rainfall breadth, temperature breadth, biome occupancy	Height and SLA	Australia	^105^
*Acacia longifolia*	20 traits	Shoot elongation	Europe	^28^
*Prunus serotina*	12 traits	RGR, LAR, A_L_, high plasticity in light energy partitioning	Europe	^106^
13 *Pinus* species	8 traits	RGR and SLA	Southwest USA	^107^
12 tree and 8 shrub species	SLA, CC, *A*, leaf nutrient concentration	SLA and *A*	Hawaii, USA	^108^
4 tree species	12 traits	RGR, LAR and *A*	Hawaii, USA	^109^
*Rhus typhina*	12 traits	Height, LMR and *A*	North China	^110^
*Robinia pseudoacacia*	9 traits	RGR and root biomass	Northeast China	^111^
*Acer platanoides*	15 traits	*A* and WUE	Northeast USA	^112^
*Bischofia javanica*	18 traits	AI and the rate of leaf production	Japan	^113^
6 tree species	RGR, SLA, LAR, RSR, leaf nutrients	SLA and high plasticity in LAR, RSR and leaf nutrient contents	Seychelles	^114^

Note: Relative growth rate (RGR), specific leaf area (SLA), net assimilation rate (NAR), leaf area ratio (LAR), leaf mass ratio (LMR), leaf mass to area ratio (LMA), total leaf area (A_L_), net CO_2_ assimilation (*A*), leaf tissue construction cost (CC), water use efficiency (WUE), acclimation index (AI) and root:shoot ratio (RSR).

Meta-analysis is one of the valuable methods to compare the growth performance of non-native and native mangroves^40^. Trait variation and plasticity comparisons between native versus non-native plants have been mostly studied in invasive herbaceous plants using meta-analyses^9,41–43^. However, woody invasive species were rarely examined (see Erskine-Ogden *et al*. 2016 for Mediterranean tree species) and, particularly no mangrove species were included in these meta-analyses. Although there are meta-analyses on mangrove productivity^44^, economic value^45^ and carbon budget of mangroves^46^, there are no meta-analyses on the growth performance of mangrove species in the existing literature.

There are only a few number of experimental studies simultaneously compared the trait responses of both native and non-native mangrove species although biological invasion in mangroves had gained a lot of attention. Some studies focus on field surveys of non-native mangrove species, whereas others measured the response of either native or non-native mangrove species^47–52^. Quantifying growth differences between native and non-native species pairs under climate change is a knowledge gap in the literature. To address this knowledge gap, we compared the growth response of co-occurring native and non-native mangrove species across different regions by examining several key performance traits in a meta-analysis. We specifically asked: Do introduced non-native mangrove species express better growth performance than co-occurring native mangrove species in their introduced regions? Is there a latitudinal gradient in growth difference between non-native and native mangrove species? Moreover, we examined the current global status of introduced species in mangrove habitats and possible mangrove responses to climate change.

## Methods

### Literature search and study selection criteria

Quantitative and qualitative data were extracted from the literature using ISI Web of Science and China Knowledge Resource Integrated Database (CNKI) in 2019. Several keywords were used to find the most related papers for our meta-analysis including mangrove*, native*, naturalized, indig*, non-native*, nonnative*, alien*, nonindig*, non-indig*, exotic*, invasive* and combinations of these keywords. Following criteria were selected to be included in the analysis: (i) Comparative empirical studies must have at least two co-occurring mangrove species (e.g., a native and non-native species). (ii) Each study must measure at least one trait (either morphological or physiological trait). (iii) Each study must be at the species-level (community-level studies were excluded). “Native” or “non-native” groups were determined based on the species information presented in each publication. For example, *Sonneratia caseolaris* is native to Hainan, China but introduced to other regions in China^53^. Therefore, it was regarded as a native species in Hainan but non-native in Shenzhen, China.

In each study, a non-native mangrove species was paired with a co-occurring native mangrove species which was presented in the same study. In the case of several native and non-native species in a single study, several pairs were included in our analysis. These species pairs were regarded as independent data points^43^ as each study has a unique response ratio including biological and environmental variation^54^. The use of co-occurring species pairs, instead of different species from separate studies, provides more realistic comparisons between non-native and native mangrove species because the data will be from the same experimental conditions (e.g., the same treatment and trait response across species).

### Extracted data and effect size

Quantitative data belonging to several traits, representing the growth and photosynthetic performance of mangrove species, were extracted (e.g., relative growth rate**–**RGR, tree height, specific leaf area, net photosynthetic rate) from tables, figures and Supplementary Files/appendices (if there are any) as these traits can be predictors of invasions^55–57^ (see also Table 2). The measuring tool in PDF-XChange Editor was used to detect values inside figures. Extracted data included species names, the origin of species (latitude), experiment type (field, survey, or greenhouse), treatment type (e.g., inundation, light, or salinity) and experimental conditions (e.g., habitat). To prevent pseudo-replication and include non-biased effect sizes, we extracted only one morphological and one physiological trait data from studies which met the criteria, and we preferably selected growth performance traits (e.g., RGR and net photosynthetic rate).

Log response ratio (LRR), that is widely used in meta-analysis studies, was preferred as an effect size to quantify the overall differences between growth differences between species pairs^58^:

1 $${\rm{LRR}}=\,\mathrm{Ln}({{\rm{X}}}_{{\rm{non}}}{/{\rm{X}}}_{{\rm{nat}}})$$

where X_non_ is the mean value in a growth trait of a non-native mangrove species and X_nat_ is the mean trait value in a growth trait of a native mangrove species.

The use of LRR allows growth performance comparison between species pairs to assess which species (non-native or native) performs well in particular environmental conditions (e.g., under high salinity or low light). If the overall value of LRR is greater than zero (lower 95% confidence interval), growth performance in non-native woody species is greater than that in native species and vice versa. If LRR overlaps zero, there is no overall growth difference between non-native and native species.

### Phenotypic plasticity calculation

In a subset of our dataset, we also used a plasticity index (PIv)^59^ to quantify phenotypic plasticity values in growth performance traits (the same traits used for the LRR) of native and non-native mangrove species pairs in China:

2 $${\rm{PIv}}=[{\rm{Max}}({{\rm{X}}}_{{\rm{c}}},{{\rm{X}}}_{{\rm{t}}})-\,{\rm{Min}}({{\rm{X}}}_{{\rm{c}}},{{\rm{X}}}_{{\rm{t}}})]/{\rm{Max}}({{\rm{X}}}_{{\rm{c}}},{{\rm{X}}}_{{\rm{t}}})$$

where X_c_ is the trait mean under control treatment; X_t_ is the trait mean under a treatment (e.g., salinity or light). PI_V_ is a common index that allows plasticity comparisons across species by illustrating the overall absolute change in trait means^60,61^. PIv values are always between 0 and 1 (representing no plasticity and maximum plasticity, respectively).

### Statistical analyses

A two-way ANOVA was used to determine the effect of trait type, experiment type and their interaction on trait responses of mangrove species. In a Tukey’s honestly significant difference (HSD) test, significant differences between trait, experiment type and their interaction were detected. In a paired sample t-test, non-native mangrove species were paired with a native species in each study and *P*-values were calculated at 0.05 level. Regression analyses were used to determine the relationship between the mean log response ratio (LRR) values and latitudes. All statistical analyses were performed in JMP software v.13.2 (SAS Institute, NC, USA). Figures were prepared using SigmaPlot v.12 (Systat Software, CA, USA).

## Results

### Dataset and trait responses in mangroves

We extracted data from four non-native mangrove species belonging to two families (33 studies) which met our selection criteria to compare the growth performance of co-occurring native and non-native mangrove species. In our dataset, there were 220 data points including nine traits, seven treatment types and 74 different experimental conditions (Appendixes A and B). In each study, non-native mangrove species were matched with a co-occurring native mangrove species. In total, there were 21 different combinations of non-native versus native mangrove species comparisons (*Kandelia candel* in China is recognized as *Kandelia obovata*^13,62^) (Appendix C).

**Figure 1:**
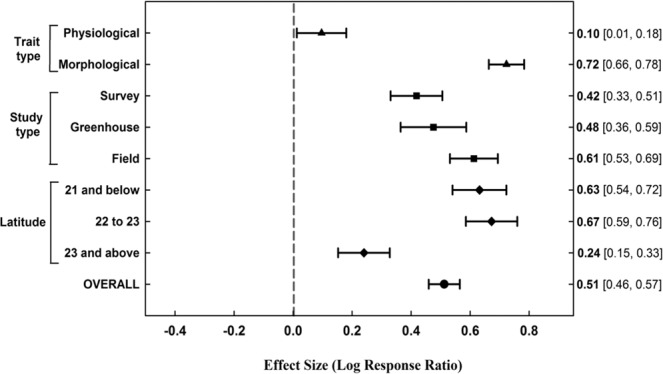
Forest plot indicating log Response Ratio (LRR) values and their 95% bootstrapped confidence intervals (CIs) across different subgroups and overall data-set (N = 220). Trait types indicate physiological traits (n = 74) and morphological traits (n = 146). Study types are field surveys (n = 79), greenhouse experiment (n = 49) and field experiment (n = 92). Latitude categories are 21°N and below (n = 69), 22 to 23°N (n = 76) and 23°N and above (n = 75).

We found a significant, positive LRR value (0.51 ± 0.05) across all studies (Fig. 1 and Appendix A). That is, recently introduced non-native mangrove species expressed significantly higher values in growth-related morphological traits (e.g., RGR and leaf area) compared to co-occurring mangrove species. The growth performance difference between these species was much more pronounced in morphological traits than in physiological traits (Fig. 1). Similarly, in field and greenhouse experiments this difference was more apparent than in field survey studies. Moreover, in high latitudes (above 23°N), the effect size was much lower than in other latitude categories.

### Effect of trait and experiment types on mangrove responses

A two-way ANOVA test indicated significant differences between trait types and trait by experiment interaction (Appendix D). There were strong differences between studies comparing non-native and native mangrove species in terms of trait types and trait by experiment interaction (Appendix D). There were no greenhouse studies using mangrove species collected from low latitudes (less than 21°N) and therefore a three-way ANOVA test was not possible to run for further analyses. Different Tukey’s HSD tests indicated that morphological traits are significantly greater compared to physiological traits (Appendix E). In survey studies, LRR values were lower than in field and greenhouse experiments but the difference was not statistically significant. Moreover, greenhouse experiments measuring morphological traits found much higher values than greenhouse experiments measuring physiological traits.

**Figure 2:**
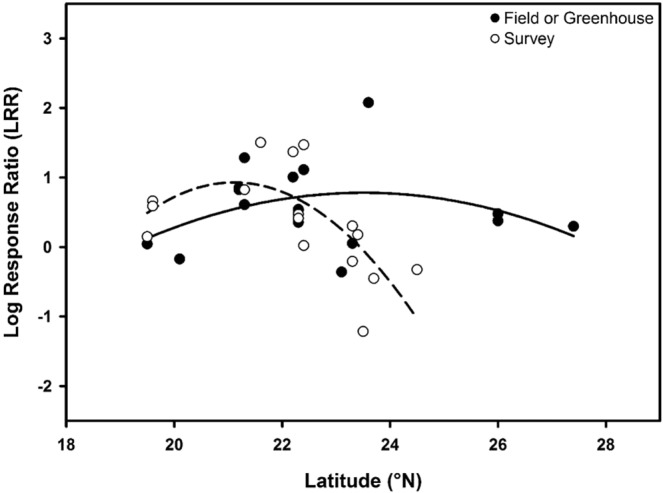
Regression analysis of mean log response ratio (LRR) values at the species level across latitudes in South China mangrove forests. The solid line indicates the regression line of field or greenhouse experiments (R^2^ = 0.12) and the dashed line indicates the regression line of survey studies (R^2^ = 0.48).

At the species level, the highest mean LRR values were in *Lumnitzera racemosa* which was introduced to Florida, USA (Table 3), followed by *Laguncularia racemosa*, *Sonneratia apetala* and *S. caseolaris*, respectively. In the dataset, most data points belonged to *Sonneratia apetala* which has been well-studied in South China. We also examined mean LRR values through regression analysis at the species level across latitudes in South China by taking averages of LRR values for each species in each location and experiment type (Fig. 2). As survey studies provide more realistic data from the field, experiment types were presented separately in the figure (survey versus field and greenhouse experiments). Interestingly, as the latitudinal gradient increased, the growth difference between non-native and native mangrove species tended to decrease in survey studies (Fig. 2).

**Table 3:** Descriptive statistics for the mean log response ratio (LRR) values at the species level.

Non-native species	N	LRR Mean	LRR SE
*Laguncularia racemosa*	44	0.53	0.12
*Lumnitzera racemosa*	24	0.57	0.19
*Sonneratia apetala*	127	0.51	0.07
*Sonneratia caseolaris*	25	0.45	0.07
Total	220		

Note: N is the sample size and SE stands for standard error.

### Trait plasticity comparison between native and non-native mangroves

We compared phenotypic plasticity of recently introduced *L*. *racemosa* and *S*. *apetala* with that of native species using a subset of our dataset (Appendix F) which contains only empirical studies that conducted a control and treatment on co-occurring mangrove species in China. Plasticity index values (PIv) in native species were calculated using arithmetic means of different native species presented in each study (e.g., *Laguncularia racemosa* versus *Rhizophora stylosa* and *Kandelia obovata*). We found that, on average, recently introduced non-native mangrove species expressed higher trait plasticity than native mangrove species in China (Table 4); however, the difference was not statistically significant according to paired sample t-test results.

**Table 4:** Paired sample t-test results of phenotypic plasticity index (PIv) for species pairs.

Species pair group	N	Mean PIv ± SE in non-native species	Mean PIv ± SE in native species	t-Ratio	Prob > |t| (*p*-value)
*Laguncularia racemosa* versus native species	16	0.41 ± 0.04	0.35 ± 0.06	1.15	0.27
*Sonneratia apetala* versus native species	32	0.46 ± 0.05	0.40 ± 0.05	1.22	0.23

Note: In each species pair group, a non-native species (e.g., *L. racemosa* or *S. apetala*) was matched with co-occurring native mangrove species. N is the sample size and SE stands for standard error.

## Discussion

### Performance of invasive and native mangrove species

High trait values in relative growth rate (RGR) and specific leaf area (SLA) are features of invasive tree species in the Mediterranean climate and these traits can be predictors of biological invasions^56^. In this study, we selected such key growth performance traits to assess the invasiveness potential of introduced mangrove species. Our results indicated that non-native mangrove species expressed higher values in growth performance traits (including RGR, height and photosynthetic traits) compared to co-occurring native mangrove species (LRR = 0.51 ± 0.05; Fig. 1). Therefore, these introduced mangrove species can be potentially invasive as a higher competitive advantage can favor biological invasions through better ability to benefit from fluctuating resource availabilities triggered by climate change (e.g., invasive tree species and associated traits listed in Table 2).

Invasion of new habitats (e.g., mangroves) depends on how fast populations can adapt to changing environmental conditions^63^. The competitive ability of non-native species can determine their invasion success^39^. As taller trees can intercept more light, tree height affects the competitive ability. If non-native species grow faster and perform better than co-occurring native species do by expressing higher phenotypic plasticity^43^, they can outcompete and even replace existing native mangrove species as global environmental changes can favor the spread of invasive plants^9^. In the case of non-native *S. apetala* invasion, forest structure can be significantly affected after the invasion and mangrove ecosystem functioning (e.g., carbon accumulation) may be changed^64,65^.

In this meta-analysis, most of the data points were from two recently introduced mangrove species (*Sonneratia apetala* and *Laguncularia racemosa*) in China. *L. racemosa* was introduced from La Paz, Mexico where is in an arid climatic zone and freshwater availability can increase its growth and productivity^66^. In Gulf of Mexico, low rainfall and high salinity prevent the movement of mangroves to higher latitudes^67^. However, at its site of introduction (Hainan, China), the mean annual rainfall is almost 9 times higher and salinity is 3 times lower than in La Paz, Mexico^68–70^. *L. racemosa* can outcompete native mangrove species (*Rhizophora apiculata*) in Hainan and it is reported to be spreading outside afforestation sites^16^. Moreover, *L. racemosa* can express high trait plasticity under environmental stresses including salinity and shade^71^. Therefore, *L. racemosa* can shift its range towards higher latitudes in China where salinity is lower and become potentially invasive owing to fast-growing characteristics, high germination rate, buoyant propagules, cold resistance, the ability to form dense monospecific stands^70,72^. These characteristics make *L. racemosa* a model species for afforestation but invoke questions regarding its invasive potential as it has frequently been used in mangrove afforestation since 2002 in China.

*Sonneratia apetala* is also an introduced fast-growing pioneer species (from Bangladesh) that can replace native species through competitive exclusion as it can grow better than native species^73^. In recently reforested areas in China, introduced *S. apetala* occupies approximately 50% of the replanted mangrove areas^13^. Moreover, the efficiency of *S. apetala* in using photosynthetic energy is higher than that in native mangrove species which can make the species invasive in South China^74^. Two mangrove species from the Indo-Pacific region planted in the US in 1940 have been invading tropical Atlantic forests^12^ and the same situation might be the case for *L. racemosa* in China whose origin is Atlantic East Pacific biogeographic zone.

When an invasive species is introduced to a new habitat, existing native plants may be more vulnerable to allelopathic chemicals originating from invasive species^75^ because such biochemicals produced by a mangrove species can prevent the germination, growth and survival of other mangrove species^76^ and therefore, determine mangrove forest succession (e.g., *Kandelia* species^77^). For example, allelopathic properties of introduced mangrove species such as *L. racemosa* and *S*. *apetala* may reduce the germination rate of seeds belonging to native mangrove species^78^ and therefore, affect the mangrove ecosystem functioning. As our meta-analysis indicated introduced mangrove species can outperform native mangrove species in their introduced regions, the invasive potential of non-native mangroves should be considered during mangrove afforestation and conservation management.

### Mangrove responses to climate change

We found that there might be a negative correlation between growth performance differences in mangroves and the latitudinal gradient in China (Fig. 2). In higher latitudes, the growth difference between non-native and native mangrove species becomes ambiguous as native mangrove species seem to perform as well as non-native species. One of the reasons behind this trend might be the adaptation to low temperatures in winter^79^. At high latitudes, naturally occurring native mangroves are expected to perform better than recently introduced species because biochemical adaptations to colder temperatures would be necessary^80^, and these adaptations can be costly for non-native species in terms of competitive ability^81^. However, as species at high latitudes experience a reduced number of freezing events as a result of climate change, cold adaptation in native species may be maladaptive.

Mangrove species expressing high phenotypic plasticity are expected to be favoured under global climate change because plasticity can allow cold tolerance and being widespread^82^. We found that introduced mangrove species in China (*S*. *apetala* and *L*. *racemosa*) express higher plasticity than co-occurring native species do (Table 4). Therefore, they might be more successful in adapting to changing environmental conditions. Therefore, findings of this meta-analysis can also predict the distribution of mangrove species and possible range shifts in mangrove species under climate change.

If certain native mangrove species cannot express appropriate performance under changing environmental conditions, they might become extinct or move to different habitats to alleviate the detrimental effects of global climate change^32,83^ because mangroves can be highly sensitive to changes in climate^84^. Global climate change-induced cyclones, tsunamis, heat waves, and climatic extremes cause significant changes in mangrove mortality and recovery^85^. Such events can reshape species composition and species abundance within mangrove ecosystems, and understanding mangrove responses to these changes is essential for mangrove ecosystem services.

### Potential implications for forest management

This study is the first meta-analysis in the literature on the growth responses of mangrove species and it has far-reaching implications for mangrove management (e.g., conservation and restoration). Interestingly, only a very limited number of studies carried out experiments using multispecies including non-native and native mangrove species pairs although several woody species were introduced to different mangrove ecosystems across the world (Table 1). Most studies on mangroves in the literature report the presence of invasive species (e.g., references in Table 1). Therefore, findings of this meta-analysis may be somewhat limited by the lack of available data and low sample size (e.g., low R^2^ in Fig. 2). However, these findings can address the situation of mangroves in South China. Due to the use of non-native mangrove species (e.g., *S. apetala*) in afforestation in the past 30 years, the invasiveness of such species has been detected using either surveys or experiments^13,53^. The findings of our meta-analysis suggest the invasive potential of non-native mangrove species based on their superior growth performance compared to native species.

The relationship between growth performance at early life stages and invasiveness can be evaluated by the probability of dominance and dispersal which are two important aspects in predicting non-native species invasion in forests^86^. Most data in the present meta-analysis were from experimental comparisons on the growth and stress-tolerance of native and non-native species pairs, reflecting the early establishment of plants. According to our effect size (LRR), these non-native species performed better during the early establishment stages. Once they survive and dominate new habitats, they can be potentially invasive.

The early life stage is also an appropriate period to reduce the biological invasions by manipulating the degree of disturbance or productivity in forest management^87^. Shading, inundation or harvesting is the major measurements to control the invasiveness of a species^88,89^. Understanding how the introduced invasive and native species respond to changing environments will be a necessity for humankind to preserve robust ecosystem functioning. Therefore, in the future, more empirical studies are needed to better examine mangrove responses to biological invasions, particularly under global climate change.

The findings of this meta-analysis can be useful to predict the distribution of mangrove species and possible range shifts in mangrove species under climate change. On the global scale, climate change is a major challenge for woody plant invasion management which might accelerate the dispersal ability and growth performance of non-native species^90^. Our meta-analysis also examined stress tolerance to several environmental factors, such as inundation, salinity, light and temperature (Appendix A). As positive LRR values indicate, better performance of current non-native mangrove species can possibly lead to invasion. For instance, the poleward range limit of non-native *S. apetala* in China is around 28°N without reproduction, whereas it is 26.5°N with reproduction^91^. On the contrary, if certain native mangrove species cannot express better performance than non-native species (low LRR value) under changing environmental conditions, they might go extinct or move to different habitats to alleviate the detrimental effects of global climate change.

Our study also draws attention to the use of non-native mangrove species in afforestation practices and mangrove conservation management across the globe because introduced non-native species can outperform native mangrove species in their introduced ranges. We suggest that the invasion potential of non-native species should be considered and the use of introduced non-native mangrove species should be avoided in mangrove afforestation practices. Although ecological repercussions of non-native mangrove species invasion in mangrove forests are uncertain, such introduced non-native species should be monitored for their potential spread. Restoration and management of mangroves are crucial for socio-economic and scientific aspects which can be achieved through a better understanding of how mangrove species respond under global climate change and biological invasion. More empirical studies in the future can enhance our understanding of mechanisms behind invasions and possible methods to control the invasion in mangrove forests.

## Supplementary information

Appendix A

Appendix B

Appendix C

Appendix D

Appendix E

Appendix F
