# Supplementary material for: Introduced non-native mangroves express better growth performance than co-occurring native mangroves
Source: Sci Rep. 2020 Mar 2;10:3854. doi: 10.1038/s41598-020-60454-z (PMC7052255; doi:10.1038/s41598-020-60454-z)
Supplement: Supplementary file 2 — Appendix B [file 41598_2020_60454_MOESM2_ESM.pdf]

Introduced non-native mangroves express better growth performance than co-occurring native mangroves

Fatih Fazlioglu[1,2] and Luzhen Chen[1*]

[1] Key Laboratory of the Ministry of Education for Coastal and Wetland Ecosystems, College of Environment and Ecology, Xiamen University, Xiamen, Fujian 361102, China

[2] Faculty of Arts and Sciences, Department of Molecular Biology and Genetics, Ordu University, Ordu, 52200, Turkey

[*] Corresponding author: Luzhen Chen

Email: luzhenchen@xmu.edu.cn

Appendix B: List of studies used in this meta-analysis.

Chen L., Peng, S., Li, J. et al., (2012) Competitive Control of an Exotic Mangrove Species: Restoration of Native Mangrove Forests by Altering Light Availability. Restoration Ecology, 21(2), 215–223. Doi: 10.1111/j.1526-100X.2012.00892.x.

Chen L., Tam N.F.Y., Huang J., et al. (2008) Comparison of ecophysiological characteristics between introduced and indigenous mangrove species in China. Estuarine, Coastal and Shelf Science. https://doi.org/10.1016/j.ecss.2008.06.003.

Chen, G-G., Dai, C-J., Li, Y-Y., et al. (2016) Comparison of Morphological Characteristics of Mangrove Plants in Coastal Areas of Fujian Province. Journal of Anhui Agri. Sci. 44(19): 178-183. Doi: 10.13989/j.cnki.0517-6611.2016.19.060.

Chen, W-P., Zheng S-F., Li, R-C., et al. (2001) Study on Constructing Mangrove Plantations in a large scale in Panyu, Guangdong Province. Forest Research. 14: 307-314. Doi: 10.13275/j.cnki.lykxyj.2001.03.012.

Chen, Y., Liao, B., Li, M. et al., (2014) Field planting experiments of mangrove on high-salinity beaches. Journal of South China Agricultural University. 35(2): 78-85. Doi: 10.7671/j.issn.1001-411X.2014.02.015.

Cheng J. (2016) Study on mangroves afforestaton technology on deep-water mudflat at Shuidong Habor. Master thesis, Southwest University, China.

Cheng, J., Lu, Y., Chen, Y., et al. (2015) Study on mangrove growth at different locations in Shuidong Harbor. Ecological Science: 34(6): 30-35. Doi: 10.14108/j.cnki.1008-8873.2015.06.005

Chuan J., Jin-wang W., Jian Z. et al., (2011) Ecological Response of Three Mangrove Species to Intertidal Position in Zhejiang. Jour. of Zheijang For. Sci. & Tech. 31(5): 45-49. Article No: 1001-3776(2011)05-0045-05.

Dangremond E.M., Feller I.C., Sousa W.P. (2015) Environmental tolerances of rare and common mangroves along light and salinity gradients. Oecologia. 179:1187–1198. Doi: 10.1007/s00442-015-3408-1.

Du, Q. and Li, L. (2018) Temporal-spatial distribution features in the root system of individual Sonneratia apetala and Avicennia marina plants. Acta Ecologica Sinica. 38(17):6055-6062. Doi: 10.5846/stxb201710111818

Feng-Lan, L., Yang Q., Zan, Q. et al., (2011) Differences in leaf construction cost between alien and native mangrove species in Futian, Shenzhen, China: Implications for invasiveness of alien species. Marine Pollution Bulletin, 62(9), 1957–1962. Doi: 10.1016/j.marpolbul.2011.06.032.

Feng-Lan, L., Yang, L., Zan, Q.-J. et al., (2017) Does energetic cost for leaf construction in Sonneratia change after introduce to another mangrove wetland and differ from native mangrove plants in South China? Marine Pollution Bulletin, 124(2), 1071–1077. https://doi.org/10.1016/j.marpolbul.2017.02.056.

Feng-Lan, L., Zan, Q.-J., Hu, Z.-Y, et al., (2016) Are Photosynthetic Characteristics and Energetic Cost Important Invasive Traits for Alien Sonneratia Species in South China? PLOS ONE, 11(6), e0157169. Doi: 10.1371/journal.pone.0157169.

Guo J., Chen X., Bao H. et al., (2016) Photosynthetic and physiological responses of mangroves under an environmental deterioration. Acta Physiol Plant. 38:140. Doi: 10.1007/s11738-016-2157-z.

Han, S-M., Li, N-Y., He, P. et al., (2010) Photosynthetic Characteristics of Introduced and Indigenous Mangrove Seedlings in China. Acta Bot. Boreal. -Occident. Sin. 30(8): 1667-1674. Article No: 1000-4025(2010)08-1667-08.

He, Z., Peng, Y., Guan, D., et al. (2018) Appearance can be deceptive: shrubby native mangrove species contributes more to soil carbon sequestration than fast-growing exotic species. Plant Soil. 432:425–436. Doi: 10.1007/s11104-018-3821-4.

Huang, M-S., Du, X-N., Liao M-M., et al. (2012) Photosynthetic characteristics and water use strategies of coastal shelterbelt plant species in Southeast China. Chinese Journal of Ecology. 31(12): 2996-3002. Article No: 1000 -4890(2012)12-2996 -07.

Jin, C. (2011) Study on Ecological Responses of Artificial Mangrove to Key Environmental Factors in Zhejiang Province. Master thesis, Beijing Forestry University, China.

Jing, Y-X., Ren, Y-L., Chen, G-Z. (2005) Studies of eco-physio logical characteristics of three mangrove species in constructed wetland sewage treatment system. Acta Ecologica Sinica. 25(7): 1612-1619. Article No: 1000-0933(2005)07-1612-08.

Li M. et al. (2012) Physiological Responses of Five South China Coastal Wetland Plants to Artificial Saline Wastewater. Journal of Anhui Agri. Sci. 40(27):13441-13504. Doi: 10.13989/j.cnki.0517-6611.2012.27.010.

Liao Y., Chen G. (2007). Research on physiological adaptability of three mangrove species to salt stress. Acta Ecologica Sinica. 27(6): 2208-2214. Article No: 1000-0933(2007)06-2208-07.

Lu, W., Yang, S., Chen, L. et al. (2014) Changes in Carbon Pool and Stand Structure of a Native Subtropical Mangrove Forest after Inter-Planting with Exotic Species Sonneratia apetala. PLoS ONE 9(3): e91238. doi: 10.1371/journal.pone.0091238

Lunstrum A. and Chen L. (2014) Soil carbon stocks and accumulation in young mangrove forests. Soil Biology & Biochemistry 75: 223-232. Doi: 10.1016/j.soilbio.2014.04.008

Miao, S., Li, D-N., Deng, H-Y. et al., (2011) Comparison of some eco-physiological characteristics of three mangrove species in Nansha District, Guangzhou. Journal of Guangzhou University. 10(1): 31-36. Article No: 1671-4229(2011) 01-0031-06.

Peng, D., Chen L., Pennings et al. (2018) Using a marsh organ to predict future plant communities in a Chinese estuary invaded by an exotic grass and mangrove. Limnol. Oceanogr. 00, 2018, 1–11. doi: 10.1002/lno.10962

Peng, Y., Diao, J., Zheng, M., et al. (2016) Early growth adaptability of four mangrove species under the canopy of an introduced mangrove plantation: Implications for restoration. Forest Ecology and Management, 373: 179-188. Doi: 10.1016/j.foreco.2016.04.044.

Tang, M., Li, K., Xiang, H. et al., (2014) Research on ecological, physiological and morphological adaptability of two mangrove species to salt stress. Ecological Science 33(3): 513-519. Doi: 10.3969/j.issn. 1008-8873.2014.03.018.

Wang, R., Liao B., He, X. et al., (2015) Effects of PGPB Inoculation on Five Species of Mangrove Seedlings in the Field. Journal of Northeast Forestry University. 43(1): 103-106. Doi:10.13759/j.cnki.dlxb.20141226.012.

Wang, X., Cai, J., Zhou, L., et al. (2018) The Analysis of Structural Features and Natural Diffusion of Laguncularia racemosa Community in Qi'ao Island, Zhuhai. Forestry and Environmental Science. 34(6): 66-71. Article No: 2096-2053(2018)06-0066-06.

Xiang, M., Liu, Q., Li, N-Y., et al. (2016) Comparison of ionic equilibrium and photosynthesis in introduced Laguncularia racemosa and two native mangrove species in China. Guihaia. 36(4):

387-396. Doi: 10.11931 / guihaia.gxzw201508016.

Yong, S., Tong, C., Zhuang, C., et al. (2011) Effects of cold weather on seedlings of three mangrove species planted in the Min River estuary during the 2010 winter. Acta Ecologica Sinica. 31(24): 7542-7550.

Zan, Q-J., Wang, Y-J., Liao, B-W. et al. (2001) Biomass and Net Productivity of Sonneratia apetala, S. caseolaris Mangrove man-made Forest. Journal of Wuhan Botanical Research. 19(5): 391-396. Article No: 1000-470X(2001)05-0391-06

Zeng, W-J., Liao, B-W., Chen, X-R. et al. (2008) The ecological effect of mangrove Sonneratia apetala mixed with three local mangrove species. Ecological Science. 27(1): 31-37. Article No: 1008-8873(2008)01-31-07
