# Supplementary material for: Introduced non-native mangroves express better growth performance than co-occurring native mangroves
Source: Sci Rep. 2020 Mar 2;10:3854. doi: 10.1038/s41598-020-60454-z (PMC7052255; doi:10.1038/s41598-020-60454-z)
Supplement: Supplementary file 3 — Appendix C [file 41598_2020_60454_MOESM3_ESM.pdf]

Introduced non-native mangroves express better growth performance than co-occurring native mangroves

Fatih Fazlioglu[1,2] and Luzhen Chen[1*]

[1] Key Laboratory of the Ministry of Education for Coastal and Wetland Ecosystems, College of Environment and Ecology, Xiamen University, Xiamen, Fujian 361102, China

[2] Faculty of Arts and Sciences, Department of Molecular Biology and Genetics, Ordu University, Ordu, 52200, Turkey

[*] Corresponding author: Luzhen Chen

Email: luzhenchen@xmu.edu.cn

Appendix C: Combination list of non-native woody species versus native mangrove species comparisons used in this meta-analysis.

**Non-Native Mangrove Species**	*Acanthus ilicifolius*	*Aegiceras corniculatum*	*Avicennia germinans*	*Avicennia marina*	*Bruguiera gymnorhiza*	*Excoecaria agallocha*	*Heritiera littoralis*	*Kandelia obovata*	*Pelliciera rhizophorae*	*Rhizophora mangle*	*Rhizophora stylosa*	*Sonneratia caseolaris* (in Hainan, China)
Laguncularia racemosa	0	6	0	3	3	0	1	16	0	0	14	1
Lumnitzera racemosa	0	0	8	0	0	0	0	0	8	8	0	0
Sonneratia apetala	2	36	0	12	13	2	0	44	0	0	13	5
Sonneratia caseolaris (in Shenzhen, China)	0	7	0	5	6	1	0	6	0	0	0	0

Note: Each species' origin and invasive status was detected from the respective publications. Numeric values indicate the number of data points in this meta-analysis. *Kandelia candel* (L.) Druce is recognized as *Kandelia obovata* (Sheue et al. 2003; Chen et al. 2009b).
