# Supplementary material for: Introduced non-native mangroves express better growth performance than co-occurring native mangroves
Source: Sci Rep. 2020 Mar 2;10:3854. doi: 10.1038/s41598-020-60454-z (PMC7052255; doi:10.1038/s41598-020-60454-z)
Supplement: Supplementary file 4 — Appendix D [file 41598_2020_60454_MOESM4_ESM.pdf]

Introduced non-native mangroves express better growth performance than co-occurring native mangroves

Fatih Fazlioglu[1,2] and Luzhen Chen[1*]

[1] Key Laboratory of the Ministry of Education for Coastal and Wetland Ecosystems, College of Environment and Ecology, Xiamen University, Xiamen, Fujian 361102, China

[2] Faculty of Arts and Sciences, Department of Molecular Biology and Genetics, Ordu University, Ordu, 52200, Turkey

[*] Corresponding author: Luzhen Chen

Email: luzhenchen@xmu.edu.cn

Appendix D: Two-way ANOVA results indicating the effect of trait type, experiment type, and their interaction.

**Variation source**	**DF**	**Sum of Squares**	**F Ratio**	***p*-value**
Trait	1	21.1	30.53	<.0001
Experiment	2	0.84	5.32	0.43
Trait × Experiment	2	4.82	12.19	<.001
