# Supplementary material for: Introduced non-native mangroves express better growth performance than co-occurring native mangroves
Source: Sci Rep. 2020 Mar 2;10:3854. doi: 10.1038/s41598-020-60454-z (PMC7052255; doi:10.1038/s41598-020-60454-z)
Supplement: Supplementary file 5 — Appendix E [file 41598_2020_60454_MOESM5_ESM.pdf]

Introduced non-native mangroves express better growth performance than co-occurring native mangroves

Fatih Fazlioglu[1,2] and Luzhen Chen[1*]

[1] Key Laboratory of the Ministry of Education for Coastal and Wetland Ecosystems, College of Environment and Ecology, Xiamen University, Xiamen, Fujian 361102, China

[2] Faculty of Arts and Sciences, Department of Molecular Biology and Genetics, Ordu University, Ordu, 52200, Turkey

[*] Corresponding author: Luzhen Chen

Email: luzhenchen@xmu.edu.cn

Appendix E: Tukey's HSD test results indicating significant differences between trait, experiment type and their interaction.

Effect	Level	Column A	Column B	Column C	Column D	Least Sq Mean
Trait type	Morphological	A				0.779
	Physiological		B			0.100
Experiment type	Greenhouse	A				0.503
	Field	A				0.464
	Survey	A				0.352
Trait * Experiment	Morphological,Greenhouse	A				1.047
	Morphological,Field	A	B			0.713
	Morphological,Survey	A	B	C		0.578
	Physiological,Field		B	C	D	0.293
	Physiological,Survey			C	D	0.127
	Physiological,Greenhouse				D	-0.119

Note: Levels are not connected by the same letter are significantly different.
